# Assessment of causal link between psychological factors and symptom exacerbation in inflammatory bowel disease: a protocol for systematic review of prospective cohort studies

**DOI:** 10.1186/2046-4053-2-8

**Published:** 2013-01-23

**Authors:** Mariyana Schoultz, Iain Atherton, Gill Hubbard, Angus JM Watson

**Affiliations:** 1 Centre for Health Science, School of Nursing, Midwifery and Health, University of Stirling, Inverness, Scotland, UK

**Keywords:** Inflammatory bowel disease, Crohn’s disease, Ulcerative colitis, Psychological factors, Symptom exacerbation, Systematic review protocol

## Abstract

**Background:**

Inflammatory bowel disease is an idiopathic chronic disease that affects around 28 million people worldwide. Symptoms are distressing and have a detrimental effect on patients’ quality of life. A possible link between exacerbation of symptoms and psychological factors has been suspected but not established. Previous reviews concerned with this link had conceptual and methodological limitations. In this paper we set out a protocol that lays the foundations for a systematic review that will address these shortcomings. The aim of this review is to provide researchers and clinicians with clarity on the role of psychological factors in inflammatory bowel disease symptom exacerbation.

**Method/design:**

We will identify all original, published, peer reviewed studies relevant to the topic and published in English from inception to November 2012. The databases MEDLINE, EMBASE, CINAHL and PsychINFO will be systematically searched. The search terms will include: inflammatory bowel disease, Crohn’s disease, ulcerative colitis, psychological stress, mental stress, life stress, family stress, hassles, social stress, coping, mood disorders, anxiety and depression in sequential combinations.

Studies will be screened according to predetermined inclusion and exclusion criteria by two reviewers. We will include clinical prospective cohort studies of all human participants aged 18 years or over with a diagnosis of inflammatory bowel disease. All eligible papers will be independently and critically appraised using the Critical Appraisal Skills Programme (CASP) tool by two reviewers. Two reviewers will independently extract and synthesise data from the studies using a predefined data extraction sheet. Disagreements will be resolved by discussion between reviewers and a third party will be consulted if agreement is not reached. Synthesised data will be analysed using Bradford Hill criterion for causality. If data permits, meta-analysis will be performed.

**Discussion:**

This study will provide the most comprehensive review and synthesis of current evidence around the link between psychological factors and symptom exacerbation in inflammatory bowel disease. Results will inform clinicians in appropriate intervention development for this patient group that would reduce symptom exacerbation and therefore improve patients’ quality of life.

## Background

Inflammatory bowel disease (IBD) is an idiopathic chronic disease that affects around 250,000 patients in the United Kingdom and around 28 million people worldwide [1,2]. IBD incidence is increasing with future prevalence likely to be considerably greater than at present [3]. With no imminent prospects of cure, the need for effective symptom management is becoming ever more pressing. Part of the development of such interventions to relieve symptoms requires a better understanding as to what actually triggers those symptoms.

IBD encompasses various different conditions, with the main two types being Crohn’s disease (CD) and ulcerative colitis (UC). Both conditions are characterized by chronic inflammation of the gastrointestinal tract. Clinically, they are often considered together given their similar aetiology and symptoms, but they differ in terms of which part of the digestive tract they affect and in the nature of the inflammation that they cause [4].

The symptoms experienced by this group of patients are often distressing. They include abdominal pain, bloody diarrhoea, nutritional failure and weight loss. However, they are not limited to the gastrointestinal tract only and can also cause ocular, musculoskeletal and skin pathologies [5]. All can occur intermittently, with periods of remission and exacerbation being experienced throughout the patient’s life. The impact of these IBD symptoms can adversely affect patients’ quality of life, affecting them psychologically, socially, educationally and vocationally [6].

Evidence suggests that a high proportion of IBD patients suffer from anxiety and depression, a percentage that is more than double when compared to healthy population [7]. This observed high anxiety and depression comorbidity in IBD patients have led many researchers and clinicians to believe that there could be a causal relationship between anxiety, depression (psychological factors in general) and IBD symptoms, even more so when other chronic diseases have established such links [8-10].

The idea about possible causality between psychological factors and IBD symptoms is not new and firstly emerged in the 1930s [11]. Since, there have been a number of reviews examining the evidence concerned with the issue, and to date, their conclusions remain somewhat contradictory [12-20]. Some have concluded that psychological factors contribute to exacerbations of symptoms [15,17] while others have refuted it [14]. More recent reviews, however, are leaning towards psychological factors having an impact on IBD symptomology, but they remain controversial and unclear [12,18-20].

This lack of clarity has brought a lot of confusion [21], particularly when empirical evidence from animal studies is suggesting potentially causal mechanisms between depression and inflammation [22,23]; and around 74% of IBD patients seem to believe that psychological factors such as anxiety and depression contribute towards symptom exacerbation [24].

This potentially causal mechanism between depression and inflammation and the patient’s belief are noteworthy influences when considering why the previous reviews have arrived at contradictory findings. Methodological weaknesses of the reviews themselves and weaknesses of the studies on which they were based on are just some of the possibilities explored by previous researchers [25] as well as the conceptual limitations [16]. Both methodological and conceptual limitations have stemmed from the complexity of the disease, the difficulty in defining psychological factors and their relationship with symptom exacerbation. All of them need a careful consideration when planning and determining the objectives of a systematic review such as this one. A summary of those identified potential limitations and recommendations are as follows:

1. The aggregation problem: some studies have assumed the psychological-physical symptom relationship to be the same for both UC and CD [19,26,27];

2. Disease activity measures: different measures of disease activity have varying levels of validity and reliability. Contrasting findings may thus have resulted depending on which tools were used [12,25];

3. Definition and measurement of psychological factors: psychological factors are complex and encompass a range of aspects and degrees of severity which could each have different implications for disease symptoms [17,25]. Similarly, utilising different tools may lead to apparently contradictory findings [28];

4. Direction of causality: studies available to previous systematic reviews have been unable to disentangle whether stress causes symptoms or symptoms cause stress, which more recent studies may have addressed [17];

5. A moderation affect: psychological factors may be an important factor for some personality types but less so, or even not at all, for others [29]. Study participants will have had different degrees of coping skills with implications for the relationship between psychological factors and disease activity. Those with more effective ability to cope will potentially have been less at risk of experiencing exacerbation of symptoms [30,31].

### Previous reviews

Systematic reviews provide robust and comprehensive overviews of research findings within a specified topic area. The aim of systematic reviews, unlike the non-systematic approach of literature reviews and overviews, is to minimise bias and offer reproducibility while using scientific and transparent approach [32]. The approach is achieved by following transparent, systematic and robust procedures.

A number of previous reviews concerned with the role of psychological factors and symptom exacerbation have not followed a systematic approach [12,18]. These papers were limited because only a single database was searched and potentially important studies were missed. This limitation might have resulted in the authors arriving at misleading conclusions. Others did not provide a clear description of their methods [19,20], denying other researchers the opportunity to make judgments on their scientific robustness [12,13,18].

Some reviews have treated IBD as a single entity [17] while others analysed specifically UC patients [14] or CD patients [15]. We highlighted earlier that IBD consists of two different diseases. For most purposes they are so similar that aggregating them together makes sense [33]. However, it has been shown that patients with clinically similar disease might vary physiologically, at a molecular level [34,35], which makes its just possible that the relationship between the psychological and physical symptomology differs in individual patients. If this were the case, then the constituent conditions should be disaggregated for analytical purposes where psychological influences are being considered. The subtlety different focuses may be a reason why the papers arrived at differing conclusions and why a review is required that distinguishes between UC and CD.

### Justification

Among the criticism about methodological and conceptual limitations, there are recommendations of previous robust reviews that should not be ignored [14-17]. However, the most recent of these is now more than a decade old, a period of time during which many more studies are likely to have been carried out. Hence, that makes a clear justification for this review to fill such a literature gap.

### Causality

Simply reporting an association between psychological factors and symptom exacerbation in IBD is not sufficient to establish causality. To prevent misleadingly causal associations, the epidemiologist Bradford Hill proposed a number of viewpoints later used as criteria that should be considered before declaring a causal relationship truly exists [36]. To date, no systematic review examining causality between psychological factors and symptom exacerbation in IBD has explicitly applied the Bradford Hill criteria to assess the evidence supporting a potentially causal association between the two.

Hence, in this paper we set out a protocol that lays the foundations for such a systematic review that will apply the Bradford Hill criteria for causality while bypassing the limitations noted from previous studies. The outcome of this review will provide clinicians with a clear foundation on which they might be able to develop therapies that reduce the likelihood of symptom exacerbation and therefore improve patient quality of life.

### Study aim and objectives

The aim of this study is to provide researchers and clinicians with clarity on the role of psychological factors in IBD symptom exacerbation. In doing so, we will conduct a systematic review that will synthesise available evidence from prospective cohort studies that are reporting on causal associations between psychological factors and symptom exacerbation in IBD and on which we will apply the Bradford Hill criteria for causality. Guided by the recommendations from previous reviews outlined in the background section, the specific objectives that will help us attain our aim are:

1. To determine whether there is a causal relationship between minor stressors and exacerbation of symptoms in IBD patients and if any causality differ between UC and CD patients;

2. Whether there is a causal relationship between life events and exacerbation of symptoms in IBD patients and if any causality differ between UC and CD patients;

3. Whether there is a causal relationship between personality type/trait and exacerbation of symptoms in IBD patients and if any causality differ between UC and CD patients.

To address the above specific objectives we will firstly identify and then examine studies concerned with the relationship between minor stressors and symptom relapse, life events and symptom relapse, and personality and symptom relapse in all IBD population. We will then synthesise and evaluate data against Bradford Hill criteria for causality and do meta-analysis if deemed appropriate.

## Method/design

### Study method

To ensure the methodology of this systematic review is robust, we will follow the 27 checklist of PRISMA statement and the guidance outlined by the Centre for Reviews and Dissemination (CRD) [37,38]. Following this guidance would ensure the methodological limitations of previous reviews outlined in the background section will be avoided.

### Criteria for considering studies for this review

#### Inclusion and exclusion criteria

To be included in the review, the papers will have to meet the following set criteria relating to study type, population of interest, risk factors studied, outcome measures and language restrictions:

##### Study types

We will only utilise prospective cohort studies that report on causal association between psychological factors and symptom relapse in IBD patients. Thus, data obtained from long-term cohort studies is of considerably higher quality to those obtained from retrospective/cross-sectional studies and retrospective cohort studies [39]. Using prospective cohort study data for the systematic review will help observe the risk factors for symptom relapse in IBD patients. Cohort studies involve observation of the individuals (over a period of time), and collection of data at regular intervals, which reduces recall error.

Cross-sectional studies are not able to ascertain the direction of effect and thus will not be included in this review. Previous reviews have acknowledged that using studies with retrospective design to answer this question could have contributed to the contradictory findings and introduced recall bias [25]. Thus we will limit our review to prospective cohort studies only.

##### Population of interest

We will include studies with all patients aged 18 years or older with diagnosis of Crohn’s disease or ulcerative colitis. Studies using mixed sample of both diagnosis will also be included. We will not use any studies using mixed sample of children and adults. While symptom presentation and therapeutic presentation could be similar between adults and children, significant differences are noted between the two populations [40].

##### Risk factors/exposure

Psychological variable specifications.

We will include studies reporting on psychological factors where they are clearly defined and the measurement tools used are clearly identified. As mentioned in the background section, psychological factors can be many and varied and often different studies will measure different psychological factors. For example, unspecified ‘stress’ will not be considered as valid psychological factor since is too vague and difficult to be accurately measured, but ‘daily stress’ or ‘perceived stress’ will be considered as valid. A sample list of clearly identified psychological factors is presented in the list below (the list is not definitive).

A sample list of Psychological factors

Anxiety

Depression

Depressive mood

Major life events/bereavement/separation

Daily events

Social stress/support

Life stress

Work/employment stress

Family stress/support

Coping

Personality

Financial stress

Satisfaction/quality of life

##### Types of outcome measures reported by studies

Disease activity and symptom relapse/exacerbation specifications

Studies reporting on disease activity and explicitly giving details on tools used to measure disease activity/symptom relapse will be included in this review. Similar to the psychological variable factors, disease activity and symptom relapse/exacerbation have to be clearly defined and measured. To be included in the review, studies will have to give details on tools used for disease activity measurement (for example, simple clinical colitis activity index -SCCAI), and details on frequency of relapses, exacerbations of symptoms and change of symptoms.

##### Language and geographical area limitations

Only studies published in English will be included in the review. Due to funding constraints, we are unable to translate studies published in other languages at this stage. There will be no geographical limitation for the included studies.

#### Search strategy for identification of studies

We will follow the guidance outlined by CRD [38] in the search and selection of studies for the review to ensure robustness. Two reviewers will independently attempt to identify all studies relevant to the review while using a pre-set screening checklist presented in Table 1. We will only include those studies that meet all criteria. The following four methods will be used to identify studies:

**Table 1:** Screening check list for inclusion to review

**Title**			
	Yes	No	Unsure
Human			
English language			
Prospective cohort study			
Reporting on psychological factors in IBD, UC or CD and disease symptoms			
Psychological variables (exposure) defined			
Disease activity and symptom exacerbation measures clearly defined			

##### Electronic searches

The database Medline will be searched via Ovid and PubMed, EMBASE via Ovid and CINAHL and PsychInfo via Ebsco will be searched for relevant articles published in English from commencement of databases to November 2012. The CRD guidance does not specify what constitutes a sufficient number of databases searched for a review as that number can vary from topic to topic [38]. Pragmatically, Medline and EMBASE might reveal most of relevant studies, however, the use of more specialised databases in addition might bring studies that are relevant to the systematic review and not included in the previous two [41].

We will use subject term services of the different databases. The following search terms and their MeSH (medical subject heading) equivalents will be used: inflammatory bowel disease, Crohn’s disease, ulcerative colitis, psychological stress, mental stress, life stress, family stress, hassles, social stress, coping, perceived stress, mood disorders, anxiety, depression, personality . These terms will be used in various combinations and wildcards will be used to pick up variant terminology. As per CRD guidance [38], a sample search strategy for Medline is presented in the list below.

Search strategy for MEDLINE

1. Inflammatory Bowel Diseases/

2. Crohn’s Disease/

3. Colitis, Ulcerative/

4. Stress, Psychological/

5. mental stress.mp.

6. life stress.mp.

7. family stress.mp.

8. hassles.mp.

9. social stress.mp.

10. coping.mp.

11. perceived stress.mp.

12. mood disorders.mp. or Mood Disorders/

13. Anxiety/

14. Depression/

15. 4 or 5 or 6 or 7 or 8 or 9 or 10 or 11 or 12 or 13 or 14

16. 1 or 2 or 3

17. 15 and 16

##### Reference lists

We will manually check the reference list of all the studies included and identified by the above search strategy to identify relevant studies that have not been detected with the database searches. These studies will be assessed against the inclusion criteria and included if appropriate. We will also check the reference list of any previous reviews on psychological factors in inflammatory bowel disease for papers suitable for inclusion in our review.

##### Citations databases

We will check Citations Google Scholar, Citations Web of Science and Citations Scopus for papers that have cited the Searle and Bennett [17] review to identify further papers that could be relevant and eligible for the review but that have not been identified using the search strategy identified above. We believe that this review is a key paper for the period up to 2001 and this paper would be referenced in more recent studies.

##### Grey literature

We will include unpublished material as part of our search given that not all relevant material will have been published. CRD recommends looking at databases such as NTIS (National Technical Information Service) and HMIC (Health Management Information Consortium) for grey literature which may reveal unpublished papers relevant to our review. We will also attempt to contact authors and experts in the field for any relevant materials rough the British society of gastroenterologists (BSG) or the World Gastroenterology Organisation (WGO). An updated search will be conducted immediately prior to data synthesis.

#### Data collection and management

##### Screening and selection of studies

All retrieved studies identified by the search strategy will be downloaded onto RefWorks and duplicates will be removed. Two reviewers will work independently. They will read title and abstract of all papers sourced to determine suitability for inclusion into the study based on the predetermined eligibility criteria (see Table 1). Discrepancies and disagreements regarding eligibility will be resolved by discussion. All papers meeting the eligibility criteria will be included for quality assessment in this systematic review. We will record reasons for exclusion of any papers excluded in quality assessment stage.

Authors will be contacted in order to clarify missing data or unclear information.

##### Data extraction and management

The two reviewers will independently extract data using a predesigned data extraction form (see Table 2). The extracted data will be grouped in general information, study characteristics, participant characteristics, setting and intervention and outcome/result data as per CRD guidance for systematic reviews [38]. Any discrepancies in extracted data will be discussed by two authors, and if consensus is not reached, a third party will be consulted. In case of incomplete data, authors will be contacted for clarification.

**Table 2:** Data extraction sheet

**General information**	
	Researcher’s name
	Date of data extraction
	Author
	Article title
	Citation
	Type of publication (for example, journal article, conference abstract)
	Country of origin
**Study characteristics**	Aim/objectives of the study
	Study design
	Study inclusion and exclusion criteria
	Recruitment procedures used (for example, details of randomisation, blinding)
	Unit of allocation (for example, participant, GP practice, and so on)
**Participant characteristic**	Age
	Gender
	Ethnicity
	Socioeconomic status
	Disease characteristics
	Co-morbidities
	Number of participants
**Setting of the study**	
**Outcome/results**	
	Whether all outcomes were defined and reported
	Measurement tool or method used
	Unit of measurement (if appropriate)
	Length of follow-up, number and/or times of follow-up
	Type of analysis used in study (for example, intention to treat, per protocol
**Conclusion as per authors**	

##### Assessment of risk of biases and methodological quality

Methodological rigour can vary from study to study and certain flaws in design or study conduct can result into bias which could influence the end result or conclusion of a study. This is particularly important for observational studies as they are often seen as at greater risk for bias.

The first step of assessing any potential bias within the eligible studies is by evaluating their methodological quality. For such evaluation the Critical Appraisal Skills Programme (CASP) tool for cohort studies will be used [42]. The CASP tool uses a systematic approach to appraise three broad areas for consideration: study validity, an evaluation of methodological quality and presentation of results and an assessment of external validity [42]. There are 12 specific questions in total assessing the following: study validity, risk of bias in recruitment, exposure, outcome measurement, confounding factors, reporting of results and the transferability of findings. Each of the questions can be answered with ‘yes’, ‘no’ or ‘can’t tell’ and each study can have a maximum score of 12.

Two reviewers will independently use the CASP tool for cohort studies and record each quality assessment. The scores will be used to grade the methodological quality of each study assessed. Discussion of unresolved disagreements regarding quality assessment with a third person will further ensure methodological rigour.

##### Data presentation

As per CRD [38], summary of extracted data from included studies will be presented in tabular form as part of the review.

##### Data synthesis, subgroup analysis and investigation of heterogeneity

We will combine data in groups by psychological factor (minor stressors, major life events and personality) with each containing three subgroups for UC, CD and mixed sample of IBD in order to address each of the aims of the systematic review (see Table 3). We will then apply the Bradford Hill criteria for causal relationship [36].

**Table 3:** Grouping of studies data for analysis

**Studies reporting on following psychological factors**	**Studies reporting on symptom exacerbation in these patient groups**	**Studies reporting on symptom exacerbation in these patient groups**	**Studies reporting on symptom exacerbation in these patient groups**
Minor stressors	UC patients	CD patients	Mixed CD and UC
Major life events	UC patients	CD patients	Mixed CD and UC
Personality	UC patients	CD patients	Mixed CD and UC

The Bradford Hill criteria are widely used to evaluate systematically whether a causal link between an exposure of interest and a health outcome exists. These criteria are often used by epidemiologists to test a causal hypothesis [43-45]. Table 4 is summarising the Bradford hill criteria for assessing causation in cohort studies together with interpretations of each criterion that will be used in this review. In addition to the Bradford Hill analysis, we will consider performing meta-analysis if appropriate. As recommended by CRD [38] and if studies characteristics are homogeneous enough, we will group studies and perform meta-analysis of the pooled data. All meta-analyses would be performed using subgroup analysis by type of disease and by type of psychological factors.

**Table 4:** Bradford Hill criteria for assessing causation in cohort studies and interpretations to be used in this review

**Criterion no.**	**Bradford Hill Criteria **[36]	**Interpretations for this review**
1. Strength of the association	The stronger the association between a risk factor and outcome, the more likely the relationship is to be causal	*For strength of association we will use odds ratio which will be graded as 1, 2, 3, 4 with 4 being strong association, 3 being moderate, 2 being weak association and 1 protective [46]
2. Consistency of findings	Have the same findings been observed among different populations, in different study designs and different times?	Findings of associations between psychological factors and symptom exacerbation have been established in other populations
3. Specificity of the association	When a single assumed cause produces a specific effect outcome	This is not going to be evaluated because single exposure to psychological factors and outcome of symptom relapse does not preclude a causal relationship
4. Temporal sequence of association	Exposure must precede outcome	Analyses will be restricted to prospective cohort studies, a design that ensures exposure will precede outcome
5. Biological gradient	Changes in disease rates should be associated with changes in exposure (dose–response)	Changes in disease (symptom) activity should correspond to changes in exposure (length or intensity of exposure to psychological factors or degree of stress experienced)
6. Biological plausibility	Presence of a potential biological mechanism of causality	Exposure selected in this review meets the criteria for plausibility of scientific credible mechanism for causality [15,17]
7. Coherence	Does the relationship agree with the current knowledge of the natural history/biology of the disease?	Current evidence needs to support an association between psychological factors and symptom relapse
8. Experiment	Does the removal of the exposure alter the frequency of the outcome?	There are experimental studies supporting the plausibility of causal relationship between psychological factors and symptom exacerbation [47]

## Discussion

There is still a debate and controversy about the influence of psychological factors in symptomology in IBD. In times when the prevalence of IBD is increasing worldwide [3,48] it is important to tackle this issue. Symptom relapse in IBD patients is often associated with worsening of quality of life [29,49]. Thus, it is important that service providers and IBD clinicians are provided with clear evidence about the relationship between psychological factors and symptom exacerbation in order to develop and implement appropriate therapies and services. Consequently, new therapies may help improve the quality of life for IBD patients by reducing factors that cause symptom exacerbation.

### Systematic review status

The systematic review is currently in the phase of screening and selection of studies. We expect completion by December 2013. PROSPERO registration number: CRD42012003143.

## Abbreviations

CD: Crohn’s disease; IBD: Inflammatory bowel disease; SCCAI: Simple clinical colitis activity index; UC: Ulcerative colitis.

## Competing interests

The authors declare that they have no competing interests.

## Authors’ contributions

MS is the lead researcher of this project. All authors contributed significantly to the design, methodology, writing and revision of the protocol. All authors read and approved the final manuscript.

## Funding

This systematic review is supported by funding from the School of Nursing, Midwifery & Health, University of Stirling.
